# Dietary zerumbone prevents mouse cornea from UVB-induced photokeratitis through inhibition of NF-κB, iNOS, and TNF-α expression and reduction of MDA accumulation

**Published:** 2011-04-06

**Authors:** Bo-Yie Chen, David Pei-Cheng Lin, Chia-Yung Wu, Mei-Ching Teng, Chi-Yun Sun, Yuan-Ting Tsai, Kuo-Chen Su, Soo-Ray Wang, Han-Hsin Chang

**Affiliations:** 1 School of Optometry, Chung Shan Medical University, Taichung, Taiwan, ROC; 2 School of Medical Laboratory and Biotechnology, Chung Shan Medical University, Taichung, Taiwan, ROC; 3 Department of Ophthalmology, Chung Shan Medical University Hospital, Taichung 402, Taiwan, ROC; 4 Department of Ophthalmology, Chang Gung Memorial Hospital, Kaohsiung, Taiwan, ROC; 5 Department of Internal Medicine, Chung Shan Medical University Hospital, Taichung, Taiwan, ROC; 6 School of Nutrition, Chung Shan Medical University, Taichung, Taiwan, ROC

## Abstract

**Purpose:**

Ultraviolet B (UVB) irradiation activates nuclear factor-kappa B (NF-κB) and inducible nitric oxide synthase (iNOS) in the cornea, resulting in inflammatory responses and malondialdehyde (MDA) accumulation. This study aims to determine the effect of zerumbone, a potent NF-κB inhibitor and inflammation modulators, on UVB-induced corneal damages in a mouse model.

**Methods:**

Fifty female imprinting control region (ICR) mice were randomly divided into five groups. The mice were anaesthetized with their ocular surfaces exposed to UVB light (0.72J/cm^2^/daily), followed by daily dietary zerumbone supplements at 0, 1, 10, and 100 mg/kg of bodyweight. Mice without zerumbone supplements were used as treatment controls and mice without UVB irradiation as blank controls. Corneal surface damages were graded according to smoothness, opacity, and the extent of lissamine green staining. Histopathological changes were also examined, along with the expression of NF-κB, iNOS, and tumor necrosis factor-α (TNF-α). MDA accumulation and the levels of two antioxidant enzymes, glutathione (GSH) and GSH reductase (GR) were also examined.

**Results:**

UVB irradiation caused significant damages to cornea, including sustained inflammation, apparent corneal ulcer, and severe epithelial exfoliation, leading to thinning of corneal epithelial layer, and infiltration of polymorphonuclear leukocytes. NF-κB expression was highly activated with nuclear translocation. The expression of iNOS and TNF-α were increased. MDA accumulation was also increased in both the corneal epithelial layer and the stroma. With dietary zerumbone, corneal damages were ameliorated in a dose-dependent manner. NF-κB activation and its nuclear translocation were blocked with decreased expression of iNOS and TNF-α. Infiltration of polymorphonuclear leukocytes was also blocked by dietary zerumbone. Besides, MDA accumulation was reduced with concomitant increase of GSH and GR levels.

**Conclusions:**

Dietary zerumbone prevents UVB-induced corneal damages by inhibition of NF-κB, iNOS, and TNF-α, with concomitant reduction of MDA accumulation and increase of GSH and GR levels in the mouse model. Results of this study suggest that dietary zerumbone may be used as a prophylactic agent against UVB-induced photokeratitis.

## Introduction

The cornea constitutes a clear front surface of the eye and is vulnerable to damages caused by UV (ultraviolet) irradiation. UV irradiation is mainly absorbed by the cornea and the anterior eye segment, through which the inner eye segments are protected from irradiation injuries [1]. Particularly, the corneal epithelium has the physiologic capacity to absorb the middle wavelength UVB (wavelength between 280 and 320 nm) and thus it acts as a UVB-filter. Despite the protective effect from cornea, excessive exposure to UVB is harmful and it represents a significant risk factor for ocular diseases. The damages caused by UV irradiation to cornea are collectively called photokeratitis, also known as ultraviolet keratitis, which is characterized by exfoliation of the corneal epithelium, reduced visual acuity, inflammation, edema, eye redness, and burning-like pain from the ocular surface [2,3]. Furthermore, the damages caused by photokeratitis may not be limited only within the corneal epithelium. UV irradiation can go deeper through the epithelial layer and induce inflammatory responses that span the full corneal thickness [4-7].

The cellular and molecular mechanisms underlying photokeratitis have been extensively investigated in recent years [8-10]. Progression of the diesease involves various proinflammatory molecules such as interleukins, cytokines, matrix metalloproteinases (MMPs) and nuclear factor-κB (NF-κB) [8,9,11-14]. Among them, NF-κB activation induced by UVB has been widely reported [8,11,15-17]. The activated NF-κB, if being translocated into the nucleus, will facilitate transcription of many downstream genes, including inducible nitric oxide synthase (*iNOS*) and cyclooxygenase-2 (*COX-2*); both are key mediators in recruitment of inflammatory cells [18-20]. Photokeratitis may also be caused by iNOS-derived nitric oxide (NO‧) production in a dose- and time-dependent manner [21,22]. The iNOS-derived nitric oxide production is activated downstream of NF-κB, followed by generation of reactive oxygen species (ROS) and other free radicals that are detrimental to cells [22]. For example, cellular lipids are easily attacked by free radicals, resulting in intracellular accumulation of malondialdehyde (MDA). As such, corneal epithelial cells are injured, leading to apoptosis.

**Figure 1:**
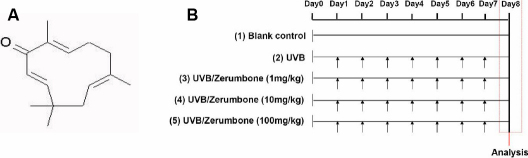
Chemical structure of zerumbone and experimental protocol for dietary zerumbone supplementation after UVB irradiation to the mouse cornea. **A**: The chemical structure of zerumbone. **B**: Daily UVB light exposure (indicated by arrows) was performed from Day 1 to Day7, with dietary zerumbone given at 1, 10, and 100 mg/kg of bodyweight, respectively, from Day 0 until Day 8. No zerumbone was given to the UVB group or the blank control group.

Zerumbone (ZER) is a sesquiterpene phytochemical, with a cross-conjugated ketone in an 11-membered ring (Figure 1A). It was found to be the main bioactive compound in the rhizome of *Zingiber zerumbet smith* grown in Southeast Asia [23,24]. It is commonly used as a condiment for food flavoring and has been shown to have antispasmodic, analgesic, antirheumatic and carminative effects in folk medicine [25,26]. Scientific researches confirmed that ZER contains many pharmacological activities, including suppression of cancer cell proliferation and downregulation of tumor invasion [24,27-29]. ZER was also shown to contain anti-inflammatory [24,26,30,31] and anti-oxidant activities [32,33], while minimally affecting normal cells [34]. In RAW264.7 macrophages treated with lipopolysaccharide or interferon-γ under in vitro conditions, ZER has been demonstrated to attenuate iNOS expression via modulation of NF-κB activation [26,35]. Thus, ZER may be potentially applied for prophylaxis against photokeratitis mediated by NF-κB. However, this potential effect of ZER has not been investigated.

In this study, we used a mouse model to test the hypothesis that ZER would ameliorate the corneal damages caused by UVB irradiation. We found that, following UVB exposure (0.72J/cm^2^/daily) for 7 days, the mouse corneas showed significant inflammatory responses and gross damages such as corneal ulcer and epithelial exfoliation. These findings were concomitant with thinning of corneal epithelial layer and infiltration of polymorphonuclear leukocytes. Besides, NF-κB, inducible nitric oxide synthase (iNOS), and tumor necrosis factor-α (TNF-α) were activated with evident MDA accumulation. With dietary ZER intake, all of the photokeratitis conditions were reversed. Our results indicate that ZER may be potentially used as a prophylaxis agent against UVB-induced photokeratitis.

## Methods

### Animals

A total of 50 six-week-old female ICR mice were purchased from National Laboratory Animal Center, Taipei, Taiwan. The mice weighted about 25 g on arrival and were fed ad libitum and kept under standard conditions with a 12-h light/dark cycle. The mice were acclimatized and habituated to the laboratory for at least one week before experiments. All mice were examined with a slit lamp (Model 99 BQ; Haag-Streit, Bern, Switzerland) before experiments. Only mice without anomalies of the anterior segment of the eye (cornea, anterior chamber, iris, or lens) were included in the experiments.

### Study groups, ultraviolet B irradiation, and ZER treatment

The 50 mice were randomly split into five groups, including (1) UVB (exposure to UVB without treatment), (2) UVB/ZER (1 mg/kg; exposure to UVB with daily ZER treatment at 1 mg/kg bodyweight), (3) UVB/ZER (10 mg/kg; exposure to UVB with daily ZER treatment at 10 mg/kg bodyweight), (4) UVB/ZER (100 mg/kg; exposure to UVB with daily ZER treatment at 100 mg/kg bodyweight), and (5) Blank control (no UVB exposure and no ZER treatment). Each group contained 10 mice. To expose the corneas to UVB irradiation, the mice were anesthetized with intraperitoneal injections of sodium pentobarbital (45 mg/kg bodyweight) and both of their eyes were exposed to daily UVB light (CN-6; Vilber Lourmat, Eberhardzell, Germany) in a darkroom. Each daily UVB exposure was performed to reach a total amount of 0.72 J/cm^2^ within 10 min. The peak wavelength of UVB light was 312 nm. The UVB light was measured using a UV detector (VLX-3W; Vilber Lourmat) from the same company. After the UVB exposure, the mice were allowed for recovery and then transferred to their original cages. The entire UVB irradiation course was completed in a consecutive 7-day period (Day 1 to Day7 in Figure 1B). For groups with ZER treatments, dietary ZER was supplemented in mouse chow, starting from Day 0 (one day before UVB exposure) and terminated on the day of analysis (Day 8 in Figure 1B). All of the ZER used in this study was purchased from Kingherbs, Inc., Hainan, China. All experiment protocols were reviewed and approved by the Animal Care and Use Committee of Chung Shan Medical University, Taichung, Taiwan and were performed in agreement with the Association for Research in Vision and Ophthalmology (ARVO) Resolution on the Use of Animals in Research.

### Scoring of corneal smoothness, opacity, and lissamine green staining

All mice were anaesthetized before assessment on Day 8 of ZER treatment. One eye of each mouse was randomly selected for assessment of corneal smoothness. The other eye was then assessed for corneal opacity. For corneal smoothness scoring, the procedures and criteria were applied following the details published by De Paiva and colleagues [36]. Briefly, images of cornea surface were taken with a stereoscopic zoom microscope equipped with ring illuminator (SMZ 1500; Nikon, Tokyo, Japan). Based on the digital images, the corneal smoothness scores were determined by using a 5-point scale based on the number of distorted quadrants in the reflected ring: 0, no distortion; 1, distortion in 1 quadrant of the ring (3 clock hours); 2, distortion in 2 quadrants (6 clock hours); 3, distortion in 3 quadrants (9 clock hours); 4, distortion in all 4 quadrants (12 clock hours); and 5, severe distortion, in which no ring could be recognized. For corneal opacity scoring, the images were scored from 0 (normal) to 4 (severe) in all corneas. The criteria were: 0, normal cornea; 0.5, mild haze seen only under dissection microscope; 1, mild haze; 2, moderate haze with visible iris; 3, severe haze with invisible iris; 4, severe haze with corneal ulceration [37]. After corneal smoothness and opacity were scored, either the right or the left eye was randomly selected and stained with 3μl of 1% lissamine green (Sigma-Aldrich, St. Louis, MO), followed by several 0.9% saline washes. Images of lissamine green staining on corneal surface were taken and scored according to a grading system based on areas of stain in the cornea [38]. Briefly, the total area of punctuate staining was designated as grade 0; grade 1, less than 25% of cornea stained with scattered punctuate staining; grade 2, 25%–50% of cornea stained with diffuse punctate staining; grade 3, 50%–75% of cornea stained with punctuate staining and apparent epithelial defects; grade 4, more than 75% of cornea stained with abundant punctuate staining and large epithelial defects. All scorings were performed by 2 observers without prior knowledge of the UVB exposure and study groups.

### Histopathological analysis and immunohistochemistry

Following assessment of corneal damages, the mice were sacrificed by cervical dislocation. One of the mouse eyes, either right eye or left eye, was randomly selected and extracted. The extracted eyes were fixed in 4% formalin for at least 24 h, washed with 0.9% saline, and processed through ethanol and xylene solutions. The preparations were then embedded in paraffin, cut at 5-µm thickness, and mounted on glass slides following conventional procedures [39-41]. Hematoxylin-Eosin (HE) stain was performed for histopathological examinations. For immunohistochemistry, the tissue sections were boiled in citrate buffer (pH 6.0) for 20 min for antigen retrieval and then incubated, respectively, with one of the following antibodies: rabbit anti-NF-kB-p65 (1/200, 1546–1; Epitomics, Burlingame, CA), or rabbit anti-iNOS (1/300, ab15323; Abcam, Cambridge, UK), or rabbit anti- Malondialdehyde (MDA) antibody (1/200, ab6463; Abcam). The preparations were then incubated with a horseradish peroxidase-conjugated secondary antibody (1/200), either anti-mouse or anti-rabbit IgG (Jackson ImmunoResearch Laboratories, Inc., West Grove, PA). After incubation, the preparations were washed thoroughly, incubated in diaminobenzidine tetrahydrochloride solution for color detection, and counterstained with hematoxylin.

### Determination of central corneal epithelial thickness

Following HE stain and histopathological examinations, the tissue sections with the longest corneal length were selected and measured for central corneal epithelial thickness under a Nikon E 100 microscope (Nikon, Tokyo, Japan). Each central corneal epithelial thickness was determined by 2 observers without prior knowledge of the UVB exposure and study groups.

### Quantification of GSH, GSH reductase, and TNF-α

In each mouse, after one eye had been randomly selected for histopathological analysis and immunohistochemistry, the other eye was used for the quantification of GSH (nmole/mg), GSH reductase (GR; U/mg), and TNF-α (pg/mg of total protein). The entire mouse corneas were isolated from the eyes under a dissection microscope and then homogenized to obtain the supernatants for analysis. The total protein level was measured by using the Lowry method. GSH concentration was measured according to the enzymatic recycling method of Anderson [42]. The amount of total GSH was determined based on a standard curve obtained with known amounts of GSH standards. GR activity was measured through the reduction of oxidized glutathione (GSSG) into GSH, which was catalyzed by GR with NADPH as the cofactor. The decrease in the optical density at 340 nm was recorded at 25 °C for 3 min and then the units of enzymatic activity were calculated using an extinction coefficient of NADPH. One unit was equivalent to the oxidation of 1 mmol of NADPH per min [43]. TNF-α was measured by using an ELISA kit **(**cat. no. CMC3010; Invitrogen, Carlsbad, CA) on a Tecan Sunrise ELISA reader (Tecan, Männedorf, Switzerland).

### Statistical analysis

All data were obtained from triple repeats and are presented as the means±standard error of the means (SEMs) and were compared among groups. The corneal smoothness, opacity, and lissamine-green staining scores were compared by Kruskal–Wallis test. The GSH and GR levels and central corneal epithelial thickness were analyzed by Mann–Whitney test. All statistical analyses were performed by using the Prism program (GraphPad Software, San Diego, CA).

## Results

### UVB irradiation causes serious damages to cornea

**Figure 2:**
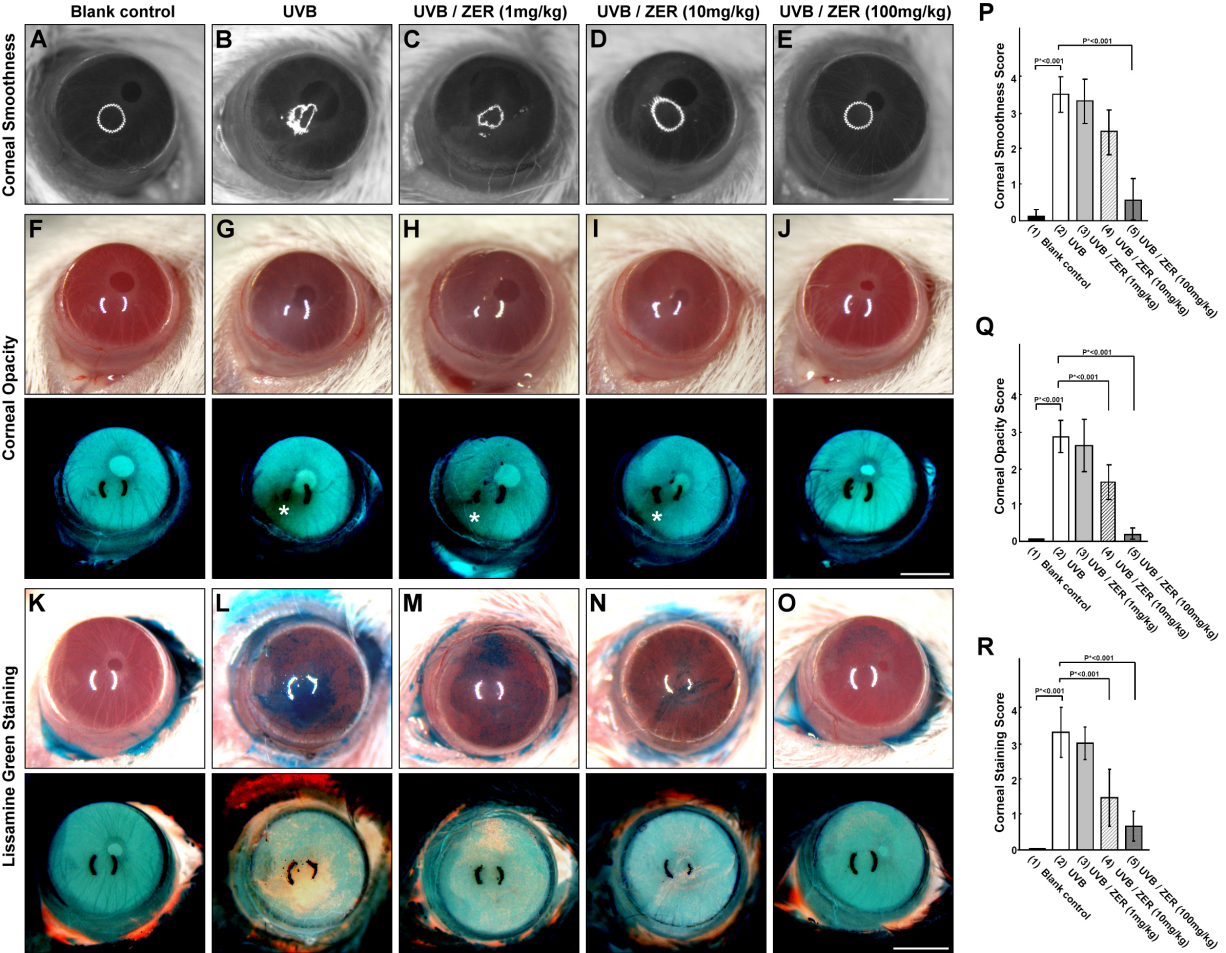
Comparison and scoring of corneal smoothness, opacity, and lissamine green staining among UVB (UVB exposure without dietary zerumbone), UVB/ZER (UVB exposure with dietary zerumbone at 1, 10, and 100 mg/kg of bodyweight, respectively), and blank control (no UVB exposure and no dietary zerumbone) groups. All scale bars: 1.25 mm.

We first examined the effects of UVB exposure on the cornea surface. The corneal phenotypes were assessed with or without topical application of lissamine green (1%) for the detection of devitalized epithelium on the ocular surface. The eyes of the UVB group generally exhibited serious damages on the corneal surface as represented by the deteriorated corneal smoothness (Figure 2B), in contrast to the blank control (Figure 2A). Quantitative analysis of corneal smoothness showed significant difference between the eyes from the UVB group and those from the blank control group (Figure 2P). Besides, corneal opacity was significantly different between the UVB group (Figure 2G) and the blank control group (Figure 2F). The difference of corneal opacity was more evidently seen in the corresponding negative images. The most severe area of opacity (marked by a white asterisk) was seen in the negative image of Figure 2G, but not in the negative image of Figure 2F. The difference of corneal opacity was also indicated by quantitative analysis (Figure 2Q). With lissamine green staining, the dark-blue devitalized epithelial areas on the ocular surface were easily identified in the eyes from the UVB group, as represented by Figure 1L and its negative image below. In contrast, no dark-blue devitalized epithelial areas were found in the eyes from the blank control group (Figure 1K and its negative image). Quantitatively, significant difference in lissamine green staining was also found between the UVB group and the blank control group (Figure 2R).

**Figure 3:**
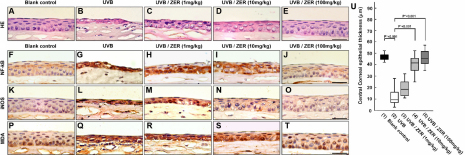
UVB-induced corneal damages, central corneal epithelial thickness, NF-κB and iNOS expression, and MDA accumulation among study groups. **A**-**E**: Hematoxylin-Eosin (HE) stain of corneas showed much thinner corneal epithelial layer with UVB exposure and the reverse effect with dietary zerumbone. **U**: The central corneal epithelial thickness was significantly reversed with dietary zerumbone, starting at 10 mg/kg. **F**-**T**: Immunohistochemical staining showed evident inhibition of NF-κB and iNOS expression with dietary zerumbone supplementation at 100 mg/kg and 10 mg/kg, respectively. NF-κB nuclear translocation in the UVB-exposed cornea in (**F**) was evidently reversed in the cornea with dietary zerumbone at 100 mg/kg in (**I**). MDA accumulation was reduced with dietary zerumbone supplementation at 100 mg/kg. All scale bars in **E**, **J**, **O**, and **T** are equal to 20μm.

**Figure 4:**
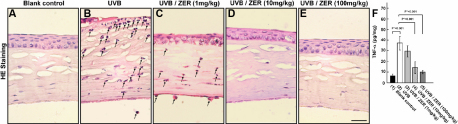
Anti-inflammatory effects of dietary zerumbone. **A**-**E**: Infiltration of polymorphonuclear leukocytes following UVB exposure, which was evidently blocked by dietary zerumbone. **F**: TNF-α expression was inhibited by dietary zerumbone. Scale bar in **E** is equal to 20 μm.

Histologically, the corneal epithelial layers with UVB exposure (Figure 3B) were significantly thinner than those of the blank control group (Figure 3A; also Figure 3G,L,Q). The epithelial cells generally exhibited more condensed nucleus, indicating occurrence of cell death (Figure 3B). Ruptures in the corneal surface were often observed in the UVB group, as exemplified by the cornea in Figure 4B). These ruptures were unlikely the artifacts due to histological processing, since the corneas from blank control group rarely showed the same damage. Furthermore, the corneas with UVB exposure commonly contained infiltrative polymorphonuclear leukocytes in the stroma (arrow-indicated in Figure 4B) and in the aqueous humor (Figure 4B,C), which was not seen in the blank controls (Figure 4A). Some polymorphonuclear leukocytes appeared to attach to the endothelial layer (Figure 4C), implying a potential risk of attack to the corneal endothelial cells.

### Dietary zerumbone repairs UVB-induced corneal damages

Many lines of evidence indicated that ZER ameliorated UVB-induced corneal damages. The scores of corneal smoothness (Figure 2P), corneal opacity (Figure 2Q), and lissamine green staining (Figure 2R) were all reduced by dietary ZER in a does-dependent manner. The mean of corneal smoothness scores was significantly reduced in the UVB/ZER (100 mg/kg) group as compared with that in the UVB group (Figure 2P; p<0.001). Significant differences in corneal opacity was also found between the UVB group and the UVB/ZER (100 mg/kg) group (Figure 2Q; p<0.001). Notably, dietary ZER at 10 mg/kg was effective in reducing corneal opacity (Figure 2Q; p<0.001). As for the reduction of lissamine green staining scores, dietary ZER at 10 mg/kg (p<0.001) and 100 mg/kg (p<0.001) were both effective (Figure 2R). Besides, dietary ZER also helped to reverse the thickness of corneal epithelial layer following UVB exposure. When compared with those in the UVB group, the eyes in the UVB/ZER (10 mg/kg) and UVB/ZER (100 mg/kg) groups were found to have thicker central corneal epithelial layer (Figure 3U; both p<0.001]. Furthermore, the infiltration of polymorphonuclear leukocytes was evidently decreased with dietary ZER in a does-dependent manner (see the reduction from Figure 4B to 4C and further to total absence in 4D). These data indicated that dietary ZER supplement at 100 mg/kg of bodyweight was most beneficial in all aspects, although ZER supplement at the dose of 10 mg/kg of bodyweight started to show positive effects.

### Dietary zerumbone inhibits UVB-induced NF-κB, iNOS, and TNF-α activation

To understand the underlying mechanisms through which ZER may repair the UVB-induced corneal damages, we performed immunohistochemical staining with an antibody against the activated p65 subunit of NF-κB to evaluate the distribution of NF-κB in the cornea. High level of NF-κB expression was seen in the corneas exposed to UVB without dietary ZER (Figure 3G), which was not observed in the corneas of the blank group (Figure 3F). Such high NF-κB expression was attenuated by dietary ZER at 100 mg/kg of bodyweight (Figure 3J) to about the same level as that of the blank control group (Figure 3F). Interestingly, high nuclear translocation of NF-κB was seen to correlate with high level of NF-κB expression (Figure 3G and 3H). As NF-κB expression was reduced, more cytoplasmic localization of NF-κB was observed (Figure 3I). When NF-κB expression was further reduced to about the same level as that in the blank control (Figure 3F), the localization of NF-κB became exclusively in the cytoplasm (Figure 3J).

To further understand how ZER may attenuate corneal inflammatory responses after UVB exposure, we examined iNOS and TNF-α expression in all of the five groups of this study. The expression of iNOS was highly induced by UVB exposure (Figure 3L), as compared to that in the blank control group (Figure 3K). In agreement with the reverse of corneal damages and inhibition of NF-κB expression, iNOS expression was repressed by dietary ZER, starting at 10 mg/kg of bodyweight (Figure 3N), with the most effective dose at 100 mg/kg of bodyweight (Figure 3O). Likewise, TNF-α expression was highly induced by UVB exposure (Figure 4F) and dietary ZER was able to reduce TNF-α expression, starting at 10 mg/kg of bodyweight, with the most effective dose at 100 mg/kg of bodyweight.

### MDA accumulation is reduced by dietary zerumbone

Since the thickness of epithelial layers was significantly reduced after UVB exposure, the epithelial cells must have been depleted beyond the corneal repair capacity. However, as the UVB-exposed corneas were analyzed on day 7, it was not explanatory to examine necrotic and apoptotic activities on the ocular surface. Therefore, we examined MDA accumulation as an alternative data to explain the repairing activity induced by ZER, as ZER had been extensively reported to contain anti-oxidant activity. Immunohistochemical staining showed that MDA was highly accumulated in the UVB-exposed cornea (Figure 3Q) in contrast to the baseline status in the blank control group (Figure 3P). With dietary ZER at 100 mg/kg of bodyweight (Figure 3T), MDA accumulation was depleted to a level close to that of the blank control group.

### Dose-dependent increase of GSH and GR with dietary zerumbone

To further elucidate the reduction of MDA accumulation in the UVB-exposed corneas, we examined the status of two anti-oxidant enzymes, GSH and GR, in the corneas of the five groups (Table 1). We found significant decrease of GSH in the corneas of the UVB group (21.42±3.44 nmol/mg protein) as compared to the blank control group (34.21±3.83 nmol/mg protein). Dietary ZER, even only given at 1 mg/kg of bodyweight, significantly increased GSH level to 25.60±5.61 nmol/mg protein (19.51% increase, p=0.093). When dietary ZER was given at 100 mg/kg, GSH level was increased to 32.65±5.20 nmol/mg protein (52.42% increase, p=0.002). Significant decrease of GR activity in the corneas of the UVB group was found, as compared to the blank control group (82.40±20.82 versus 135.34±23.92 U/mg protein). Dietary ZER, even only given at 1 mg/kg bodyweight, significantly increased GR activity to 97.60±21.75 U/mg protein (18.45% increase, p=0.172). When dietary ZER was given at 100 mg/kg, GR activity was increased to 125.32±18.70 U/mg protein (50.52% increase, p=0.003). These data support a role of dietary ZER in the depletion of MDA accumulation through GSH and GR activation in a dose-dependent manner.

**Table 1:** Antioxidant concentration (level) in cornea.

	**GSH (nmol/mg protein)**	**GR (U/mg protein)**
(1) Blank control	34.21±3.83	135.34±23.92
(2) UVB	21.42±3.44	82.40±20.82
(3) UVB/ZER (1 mg/kg)	25.60±5.61	97.60±21.75
(4) UVB/ZER (10 mg/kg)	26.18±6.74	103.37±21.37
(5) UVB/ZER (100 mg/kg)	32.65±5.20	125.32±18.70
**Percent change**		
(2-3)	19.51%	18.45%
(2-4)	22.13%	25.45%
(2-5)	52.42%	50.52%
P* (1)-(2)-(3)-(4)-(5)	0.001	0.001
P** (2-3)	0.093	0.172
P** (2-4)	0.208	0.141
P** (2-5)	0.002	0.003

Both enzymes were significantly increased with dietary zerumbone at 100 mg/kg. p*: Kruskal Wallis test; p**: Mann Whitney U test.

## Discussion

**Figure 5:**
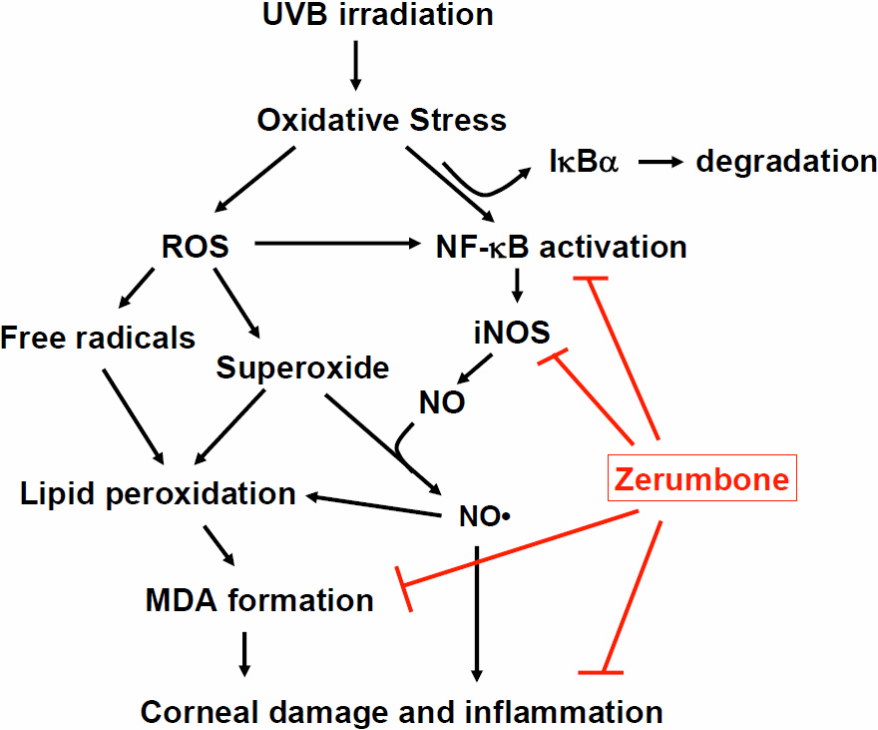
Diagrammatic illustration of the effects of dietary zerumbone against UVB-induced photokeratitis as shown in this study.

The ocular system receives lights as input signals from the environment. Therefore, UV exposure is inevitable and poses as a major risk factor for many eye diseases, including photokeratitis, climatic droplet keratopathy, cataract, pinguecula, and pterygia formation [44,45]. Particularly, UVB irradiation is most critically involved in the pathogenesis of photokeratitis, since most of its energy is absorbed by the cornea [46]. When eyes are excessively exposed to UVB, the oxidative stress will be induced to a phototoxic level, leading to transcriptional activation of inflammatory factors such as NF-κB and iNOS and formation of cytotoxic nitric oxide and nitrogen-related oxidants [47]. Eventually, the inflammatory responses will cause a variety of damages to the cornea (Figure 5).

Given that UV exposure is inevitable, potential ways to prevent against the phototoxic effects caused by UVB have been extensively studied. Physically, UV shields should be used for protection. Chemically, inhibition of inflammatory factors and depletion of reactive oxygen species (ROS) or ROS-attacked biomolecules constitute two major means to modulate the UVB-induced phototoxic effects. ZER had been widely reported to have dual anti-oxidant and anti-inflammatory activities without evident side effects to normal cells [26,30-34]. This study investigated the effects of dietary ZER on UVB-induced photokeratitis in a mouse model, since there had been no report on ZER application in this respect. Our results showed that dietary ZER acts not only to inhibit UVB-induced NF-κB, iNOS, and TNF-α expression, but also to deplete MDA accumulation. Besides, we showed that dietary ZER can increase the production of two anti-oxidant enzymes, GSH and GR, in the cornea following UVB exposure. Without prior publication in this respect, our results represent the first assessment of ZER as a potential agent for prevention against photokeratitis.

NF-κB is a pro-inflammatory factor that acts upstream of many inflammatory activators, including Cox-2, iNOS, Bcl-xl, CIAPs, and cyclin D1 under in vivo and in vitro conditions [48-50]. In the cornea, previous reports had indicated that UVB can induce NF-κB activation [51] and its nuclear translocation [11]. Despite the prior knowledge, only an NSAID COX inhibitor, namely lornoxicam, had been assessed as a candidate agent to down-regulate nuclear NF-κB in mouse corneas after UVB exposure [51,52]. Because NSAIDS typically interact with other drugs, they are not ideal for use regularly. Thus, further searches of other candidates to inhibit UBV-induced NF-κB activity are mandatory. Here, we provide the first evidence that ZER inhibits UVB-induced NF-κB activation in the mouse cornea under in vivo conditions. This inhibitory effect of ZER is further confirmed with the downregulation of iNOS, a well known downstream target of NF-κB activation [26,35] (Figure 5).

Despite our current findings, the underlying mechanism remains to be elucidated. Oxidative stress and reactive oxygen species have been shown to activate NF-κB in the mouse and the rabbit cornea [53,54]. It is likely that ZER inhibits NF-κB activation through depletion of reactive oxygen species, as reflected by the reduction of MDA accumulation and the increased expression of GSH and GR anti-oxidant enzymes. Another potential mode of action by ZER is through its inhibitory effects on Cox-2 expression, which was shown in murine macrophages [55]. UVB-induced Cox-2 expression has been confirmed in mouse skin epidermal cells [56] and Cox-2 expression is known to involve in acute ocular inflammation [57], particularly through the activation of NF-κB in the cornea [53,54]. Therefore, ZER may act through inhibition of Cox-2 expression to repress ocular inflammation. A third potential mode of action by ZER may be through direct regulation of NF-κB transcription, which remains to be investigated in future studies.

NF-κB plays a key role in the regulation of apoptosis [49]. In addition, ROS and MDA are cytotoxic to all cells. As dietary ZER can reduce NF-κB, iNOS, and TNF-α expression as well as MDA accumulation, with simultaneous increase of GSH and GR levels, the mouse corneal epithelial layers were significantly repaired. It is therefore reasonable to see the reduced thickness of corneal epithelial layer after UVB exposure and the reverse effect with dietary ZER treatment. However, whether dietary ZER could directly or indirectly alter the process of UVB-induced cell death is unknown. With UVB exposure, the downstream effectors controlled by NF-κB, iNOS, and MDA evidently predominated over the antiapoptotic effectors triggered by the cellular defensive responses. ZER is likely to act through repression of proapoptotic effectors or promotion of antiapoptotic effectors or both. Based on the data obtained in the present study, we can not assume that ZER promotes antiapoptotic effectors, but at least the downstream proapoptotic effectors controlled by NF-κB, iNOS, or MDA must have been repressed by dietary ZER, so that the thickness of corneal epithelial layer was reversed. These protective effects might also be regulated with simultaneous reduction of ROS and MDA by the antioxidant activity of ZER. Furthermore, as the corneal GSH/GR levels are increased with dietary ZER (Table 1), the protective effects might also be mediated through induction of detoxification enzyme such as GST (Gluthathione S-tranferase) by ZER, which had been demonstrated in the rat liver epithelial cell line (RL34 cells) [33].

Drug intervention to prevent UVB-induced photokeratitis has been studied in recent years. For example, lornoxicam was shown to protect mouse cornea from UVB-induced damages and to suppress recurrent herpetic stromal keratitis [51,52]. Another example is 4-coumaric acid (4-CA) that has been shown to protect rabbit corneal-derived cells from UVB-induced oxidative damages [58]. The other examples include rebamipide (an antigastric ulcer drug) and carteolol hydrochloride (an anti-hypertension drug for glaucoma); both were also evaluated to protect against UVB-induced corneal damages in mice [59]. Despite previous studies, intervention using natural food components has not been as widely studied as using drugs, although dietary food is more acceptable. ZER is traditionally used as a condiment for food flavoring [30,60] and will be more acceptable for the prevention of UVB-induced photokeratitis.

In summary, a mouse model of UVB-induced photokeratitis was successfully established and the preventive effects of dietary ZER were demonstrated in this study. Typical histopathological changes following UVB exposure are all reversed by dietary ZER, with inhibition of NF-κB, iNOS, and TNF-α expression and reduction of MDA accumulation. We conclude that ZER may be used as a prophylactic agent against UVB-induced photokeratitis.
